# Synthesis and Evaluation of Starch-Grafted-Poly[(Acrylic Acid)-*Co*-Acrylamide] Based Nanoclay Polymer Composite Fertilizers for Slow Release of Nitrogen in Soil

**DOI:** 10.3390/polym16213013

**Published:** 2024-10-27

**Authors:** Ravi Saini, Kanchikeri Math Manjaiah, Dibakar Roy, Rajesh Kumar, Sandeep Gawdiya, Siyaram Meena, A. Naveenkumar, Anil Kumar, Salah El-Hendawy, Mohamed A. Mattar

**Affiliations:** 1 Division of Soil Science and Agricultural Chemistry, ICAR-Indian Agricultural Research Institute (IARI), New Delhi 110012, India; ravisaini09121998@gmail.com (R.S.); dibakar499176@gmail.com (D.R.); siyarammeena092@gmail.com (S.M.); naveenkmr239@gmail.com (A.N.); akrajput1975@gmail.com (A.K.); 2 ICAR-National Bureau of Soil Survey and Land Use Planning, Regional Station, Jorhat 785004, India; 3 Division of Agricultural Chemicals, ICAR-Indian Agricultural Research Institute (IARI), New Delhi 110012, India; rajesh.kumar16@icar.gov.in; 4 Division of Agronomy, ICAR-Indian Agricultural Research Institute (IARI), New Delhi 110012, India; sandeepagro78626@gmail.com; 5 Department of Plant Production, College of Food and Agricultural Sciences, King Saud University, P.O. Box 2460, Riyadh 11451, Saudi Arabia; mosalah@ksu.edu.sa; 6 Department of Agricultural Engineering, College of Food and Agricultural Sciences, King Saud University, P.O. Box 2460, Riyadh 11451, Saudi Arabia

**Keywords:** nitrogen, nanoclay biopolymer composites, poly[(acrylic acid)-*co*-acrylamide], slow-release fertilizers

## Abstract

Nitrogen (N) losses from conventional N fertilizers contribute to environmental degradation and low N use efficiency. Highlighting the need for slow-release fertilizers (SRFs) to mitigate these problems, this study aims to develop slow-release N fertilizers using starch-grafted-poly[(acrylic acid)-*co*-acrylamide] based nanoclay polymer composites (NCPCs) and investigate their efficacy for slow N delivery in soil. Three types of NCPCs, NCPC(A) (poly [(acrylic acid)-*co*-acrylamide]), NCPC(W) (wheat starch-grafted-poly[(acrylic acid)-*co*-acrylamide), and NCPC(M) (maize starch-grafted-poly[(acrylic acid)-*co*-acrylamide) were prepared and characterized using FTIR spectroscopy and X-ray diffraction techniques. N-release behaviour of the products was assessed under two distinct soils, i.e., Assam (Typic Hapludults, pH 4.2) and Delhi (Typic Haplustepts, pH 7.9) soils. Additionally, the effects of varying soil moisture and temperature levels on N release were studied in the Assam soil. The N-release kinetics of the synthesized fertilizers were assessed using zero-order, first-order, Higuchi, and Korsmeyer−Peppas models. Degradability of the NCPCs was evaluated by measuring evolved CO_2_–C under various soil conditions as an indicator of microbial degradation. The results indicated that NCPC fertilizers significantly slowed down the release of N compared to urea. According to the R^2^ values obtained, it was evident that the first-order kinetic model most accurately describes the N release from both urea and NCPC-based N fertilizers in the studied soils. Among the formulations, NCPC(A) exhibited the lowest N release (42.94–53.76%), followed by NCPC(M) (51.05–61.70%), NCPC(W) (54.86–67.75%), and urea (74.33–84.27%) after 21 days of incubation. The rate of N release was lower in the Assam soil compared to the Delhi soil, with higher soil moisture and temperature levels accelerating the release. Starch addition improved the biodegradability of the NCPCs, with NCPC(W) showing the highest cumulative CO_2_-C evolution (18.18–22.62 mg g^−1^), followed by NCPC(M) (15.54–20.97 mg g^−1^) and NCPC(A) (10.89–19.53 mg g^−1^). In conclusion, NCPC-based slow-release fertilizers demonstrated a more gradual N release compared to conventional urea and the inclusion of starch enhanced their degradability in the soil, which confirms their potential for sustainable agricultural applications. However, soil properties and environmental factors influenced the N release and degradation rates of NCPCs.

## 1. Introduction

Nitrogen (N) is a key nutrient element for crop production and is considered the most important yield-limiting factor [1]. It is an important constituent of amino acids, proteins, nucleotides, chlorophyll, as well as plant hormones [2]. Among the fertilizers, crops require N in the largest quantity. The global synthetic N fertilizer consumption in the year 2018 was recorded at 104 Tg [3]. In India, the total consumption of N fertilizers has increased from 55,000 tons in 1950–51 to 20.21 million tonnes in 2022–23 [4], and the N fertilizer consumption will further increase to meet the demand for food for the ever-increasing population [5]. However, Overuse of fertilizer can lead to low nutrient use efficiency and increased production costs. Additionally, the loss of N fertilizer exacerbates agriculture’s negative environmental impact through greenhouse gas emissions, groundwater contamination, and surface water source eutrophication [6,7]. Urea is the most widely used synthetic N fertilizer due to its high N content (46%) and low price [3,8]. However, only 30% to 65% of the N applied through urea is used by the crops [9,10]. This is due to the fact that a sizable portion of the N from urea is released right away after application due to rapid hydrolysis and transformation in moist soil and is not timed with the N uptake of seasonal crops [11,12,13]. As a result of the imbalance, urea applications could experience up to 70% N losses [14], especially due to ammonia (NH_3_) volatilization and nitrate (NO_3_) leaching [15,16,17]. Globally, the supply chain of synthetic N fertilizer is accountable for 10.6% of agricultural greenhouse gas (GHG) emissions, of which 38.8% stem from N fertilizer production [18,19]. Since these well-known environmental issues brought on by excessive fertilizer use have been identified, the research and development of novel controlled and slow-release fertilizers (CRFs and SRFs) has garnered more focus over the past twenty years [20,21].

Nanoclay polymer composites (NCPCs)-based slow-release fertilizers are a new potential alternative to conventional fertilizers to increase N use efficiency and minimize the losses of N to the environment. Nanoclay polymer composites are synthesized by combining inorganic clays with synthetic polymeric superabsorbents [22]. These superabsorbent polymers have an incredible capacity to absorb and retain water and nutrients for an extended period because of their three-dimensional polymeric network structure. Poly(acrylic acid-*co*-polyacrylamide) is a copolymer superabsorbent that possesses hydrophilic properties with a large number of polar groups [23,24]. Poly(acrylic acid-*co*-polyacrylamide) based superabsorbent has recently gained a lot of attention as a water-retentive material and a carrier for the slow release of nutrients for use in agriculture [25]. These clay polymer composites are combined with the conventional N sources to develop slow-release N fertilizers, which release plant-available N gradually over an extended period [26]. However, because these superabsorbents are made of pure polymers like acrylic acid and acrylamide, they are noneconomical and have poor biodegradability in soil, which might be causing new types of contamination in soil due to the accumulation of undesirable residue of the synthetic material [27]. To improve the degradability and to produce products involving reduced cost, the use of new technologies in recent years, such as starch grafting or the addition of inorganic clay to organic-inorganic superabsorbent composites, has somewhat altered this state [27,28]. Biodegradation of acrylamide occurs as microorganisms utilize the amide group of the polymer as an N source and/or the carbon backbone as a carbon source [29]. However, it is much harder for microorganisms to utilize the carbon backbone of acrylamide as a sole carbon source [30]. Therefore, the formulation of starch-grafted polymers ensures significant improvements in biodegradability because starch provides a labile source of carbon, which is easily metabolized by soil microorganisms and improves the overall biodegradability of the NCPCs. The NCPC-based fertilizers release plant-available N gradually over an extended period of time with a greater probability of matching the crop N requirements [5]. However, how quickly the fertilizers release nutrients is greatly influenced by the soil and environmental factors [31]. When using two or more dissimilar soil types (varying in pH, texture, and clay mineral types), the N release rate from the same NCPC composite material may differ [26]. The effects of soil and environmental drivers on the release of N from superabsorbent-based slow-release N fertilizers and subsequent transformations of inorganic N in soil need to be studied thoroughly.

Therefore, The present investigation was designed with the following objectives: (i) to develop starch-grafted Nanoclay polymer composite (NCPC) based slow-release N fertilizers; (ii) to study the effect of the inclusion of natural starch in the poly[(acrylic acid)-*co*-acrylamide] NCPC matrix on the biodegradability of these polymer composites; and (ii) to investigate the effects of soil properties and environmental drivers on the N release dynamics from NCPC based slow-release N fertilizers and the subsequent transformations of inorganic N in soil. The novelty of this study lies in the successful development of starch-grafted-poly[(acrylic acid)-*co*-acrylamide] NCPC-based slow-release N fertilizers with improved biodegradability in soil and the evaluation of N release kinetics and degradation rate of the composites under diverse soil and environmental conditions.

## 2. Materials and Methods

### 2.1. Preparation of Nano Clay Polymer Composites (NCPC)

#### 2.1.1. Materials

The extra pure chemicals were used for the synthesis of the NCPCs. The chemicals and their details are as follows: acrylamide with a purity of ≥99% (CAS number 79-06-1), acrylic acid with a purity of ≥98.5%, ammonia (25% solution) with a purity of ≥25% (CAS number 1336-21-6), N,N’-methylenebisacrylamide (MBA) with a purity of ≥98% (CAS number 110-26-9), and ammonium persulfate (APS) with a purity of ≥97.5% (CAS number 7727-54-0). Bentonite was procured from Sigma-Aldrich (Bangalore, India), which had a cation exchange capacity of 84 cmol(p+) kg^−1^.

#### 2.1.2. Synthesis

**Figure 1:**
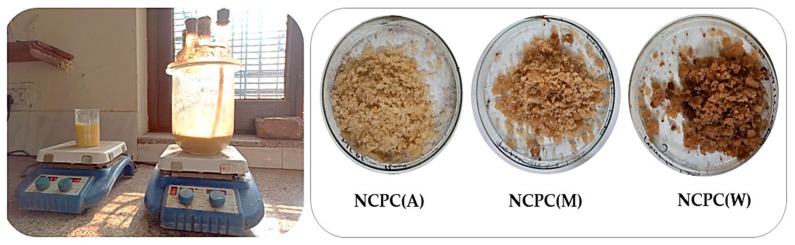
Synthesis of nanoclay polymer composite fertilizers.

**Scheme 1:**
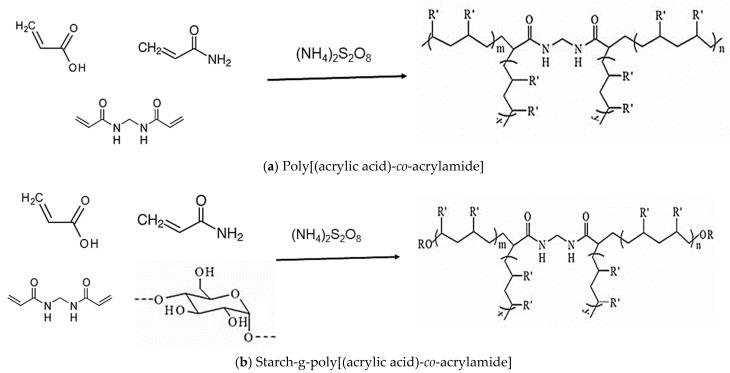
Schematic presentation of the copolymerization of acrylic acid and acrylamide (**a**) and graft copolymerization of acrylic acid and acrylamide with starch (**b**), wherein R’ represents CONH_2_ or CO_2_H.

The NCPCs were synthesized by copolymerization reaction of acrylic acid (AA) and acrylamide (AM) (see Scheme 1), along with partial neutralization with ammonia solution [32]. In order to synthesize NCPC(A) (i.e., Poly[(acrylic acid)-*co*-acrylamide]), 23.04 g of acrylic acid was neutralized with ammonia to a 60% neutralization degree in a reaction kettle equipped with a magnetic stirrer cum heater and connected with inert nitrogen gas supply. After the neutralization reaction was completed, 4.6 g of acrylamide and commercial bentonite as 10% of total Aa + Am was added to the reaction vessel. Methylene bisacrylamide (N,N MBA) (0.2308 g) and ammonium persulfate (APS) (0.3204 g) were added as a crosslinking agent and free radical initiator, respectively. The temperature gradually increased to 70 °C and allowed to complete the polymerization reaction (Figure 1). Upon completion of the polymerization process, the polymer composites were solidified, which were collected in petri plates and washed with distilled water.

For the synthesis of starch-grafted NCPCs (NCPC(W) and NCPC(M)), 20% of acrylic acid was substituted with wheat and maize flour as a starch source. The weighed quantity of wheat or maize flour with 100 mL of distilled water was taken in a 500 mL beaker, and 0.1g of APS was added. The contents were kept on a magnetic stirrer cum heater at 110 rpm and 70 °C for 30 min. After gelatinization of the starch, the contents were transferred to the reaction cattle containing partially neutralized acrylic acid and acrylamide mixture. The remaining procedure remains the same as NCPC(A) synthesis. Polymers were dried at a temperature between 60 to 70 °C. To achieve a consistent particle size, the polymers were ground and sieved through a 2 mm screen. The details of the synthesized products are given in Table 1.

A known quantity of aqueous solution of urea was prepared in distilled water for loading the polymers with nitrogen. To achieve swelling equilibrium, the dry ground gels were submerged in the solution. The gels that had swelled then dried at 60 °C until constant weight was achieved, and they had been used for additional analysis and applications.

### 2.2. Characterization of NCPCs

#### 2.2.1. Fourier Transform Infrared (FTIR) Spectroscopy

FTIR is widely used for the detection of functional groups in compounds. A specific wave number indicates the location of a given absorption band. The functional groups absorb energy from a particulate infrared region, quantizing the vibrations. Here, FTIR spectroscopy of the synthesized products was carried out using the Bruker: ALPHA, FTIR/ATR system (24 scans, resolution–4 cm^−1^) (Bruker India Scientific Private Limited, Mumbai, India. The powdered samples were scanned in the 4000–400cm^−1^ wavelength region. Then, all the spectra were analyzed to know the type and nature of the functional group attached.

#### 2.2.2. X-Ray Diffraction Technique (XRD)

XRD is a non-destructive analytical technique for identifying and quantifying various crystalline forms. When X-rays interact with a regular structure whose repeat distance is almost equal to the wavelength, diffraction occurs [33]. The XRD analyses of the synthesized products were carried out using a Philips diffractometer (X-ray generator PW 1729, diffractometer control: PW 1710, Philips, (Amsterdam, The Netherlands) using APD (automated powder diffraction) software with the start angle (°2θ)-4.00, end angle (°2θ)-50.00 and continuous type of scan.

#### 2.2.3. Nutrient Content Analysis

The total N content in the synthesized fertilizers was estimated by the Kjeldahl digestion-distillation method [34]. Fertilizers were digested with concentrated sulphuric acid + digestion mixture with the help of a digestion unit (Kelplus). After cooling, the contents of the tube were distilled with 40% NaOH using a Kjeltec distillation unit (Kelplus-Classic DX, Pelican Instruments, Tamil Nadu, India). The released ammonia was absorbed in a 4% boric acid solution and titrated with standard H_2_SO_4_. The total carbon content in the synthesized fertilizers was analyzed using a CHNS analyzer (EURO EA3000 elemental analyzer by Eurovector Elemental Analysis Company) (Table 1).

### 2.3. Water Absorption and Retention Capacity

To determine the maximum water absorbency of NCPCs, a 1 g sample of the polymer composites was immersed in the excess of distilled water and left at room temperature to reach the swelling equilibrium. After reaching the swelling equilibrium, the free water was filtered through a 200-mesh nylon sieve, and NCPCs were allowed to drain for 15 min and weighed [35]. The following equation was used to determine the equilibrium water absorbency (QH_2_O):

QH_2_O (g/g) = (W2 − W1)/W1 (1)

where W1 is the weight of NCPC and W2 is the weight of swollen NCPC. QH_2_O was expressed as grams of water absorbed per gram of sample.

After reaching the swelling equilibrium, to study the water retention capacity of the polymer composites, the weight of the swelled polymer composites was recorded at different time intervals till the polymer composite’s moisture content reached equilibrium with the atmosphere.

### 2.4. Soil Characterization

Two surface (0–15 cm depth) soils were used for the laboratory experiment: Assam soil (acidic soil, pH = 4.2, Typic Hapludults) from Jorhat, Assam and Delhi soil (alkaline soils, pH = 7.9, Typic Haplustepts) from ICAR-IARI, New Delhi, India. The soil samples were collected and processed in accordance with the standard soil sampling procedure. Samples of collected soil were examined for different physico-chemical soil properties (Table 2).

### 2.5. Nitrogen Release Study

An incubation study was carried out to study the N release pattern of synthesized products NCPCs in two different soils (Assam and Delhi). The experiment with Assam soil was comprised of four fertilizer treatments along with one unfertilized control, two incubation temperatures (25 and 35 °C), and two soil moisture levels (field capacity and maximum water holding capacity), which were replicated three times. The experiment with Delhi soil was similar but had no temperature and moisture factors; fertilizer–soil mixtures were incubated at 25 °C and field capacity only. A 150 g of soil was taken in a beaker, and 100 mg of N was added with different fertilizer sources. Fertilizer dosage was applied by putting fertilizer in a muslin cloth bag. Mineral N released in soil was analyzed after 0, 7, 14, 21, 28, and 56 days of incubation. To eliminate N from native soil, we also took a replicated beaker containing the desired volume of soil without any fertilizer product; N released from urea and urea-loaded NCPC products are obtained upon subtraction of N contributed from the soil itself. Destructive sampling was carried out during sample collection for mineral N estimation. Ammoniacal and nitrate N released in the soil were extracted with 2M KCl solution [43] and determined by distillation method [36].

The N release kinetics of all the synthesized fertilizer products, along with urea, was assessed through zero-order, first-order, Higuchi, and Korsmeyer−Peppas kinetic models.

#### 2.5.1. Zero-Order Release Kinetics

The zero-order kinetic model indicates a constant release rate over time, which can be expressed using the below equation:

(2) $$Q_{t}=Q_{0}+k_{0}t$$

where *Q_t_* is the amount of nutrient released in time *t*, *Q*_0_ is the initial amount of nutrient in the solution, and *k*_0_ is the zero-order release constant [44]. The model was examined by plotting the cumulative nutrient release (*Q_t_*) against time (*t*). The slope at which the graph is linear can be used to determine the value of the release constant, *k*_0_.

#### 2.5.2. First-Order Release Kinetics

The first-order model demonstrates the nutrient release depending on the concentration [45]. Nutrient release that followed the first-order model can be expressed using the below equations:

(3) $$C_{t}=C_{0}(1-e^{-k_{1}t})$$

the linear form of the equation is given as:

(4) $$logC_{t}=logC_{0}-k_{1}t/2.303$$

where *C_t_* is the cumulative nutrient released at time t, *C***_0_** is the amount of potentially mineralizable nutrient in the fertilizer, and *k*_1_ is the first-order rate constant [46]. The model was assessed by plotting the obtained cumulative log percentage of nutrients released against time. The graph is linear, and the slope is given by −*k*_1_/2.303.

#### 2.5.3. Higuchi Release Kinetic Model

The nutrient release from an insoluble matrix is described by the simplified Higuchi model as a square root of a time-dependent process based on Fickian diffusion, which can be expressed using the below equation:

(5) $$Q=k_{H}t^{1/2}$$

where *Q* can be represented as Mt/M∞. Mt represents the nutrients released at time *t*, and M represents those released at time infinity. *k_H_* is the Higuchi dissolution constant [47]. The model was examined by plotting the cumulative percentage of nutrient release (*Q*) against the square root of time (*t*).

#### 2.5.4. Korsemeyer−Peppas Kinetic Model

The nutrient release from the polymeric system can be described by the Korsmeyer-Peppas model. The Fickian and non-Fickian release of nutrients from swelling and non-swelling polymer systems can be ascertained empirically. The model equation (power law) can be expressed as follows:

(6) $$Q=kt^{n}$$

where *Q* can be represented as Mt /M∞. Mt represents the nutrients released at time *t*, and M represents those released at time infinity. *k* is the rate constant [48]. Log cumulative percentage nutrient release was plotted against log time to study the model.

### 2.6. Degradation Study

The degradability of NCPCs was studied by measuring evolved CO_2_–C as a time interval when the NCPC products were incubated in soil. Likewise, in the N release study, in Assam soils, we studied the degradation rate of the products at two soil moisture (field capacity and maximum water holding capacity) and two temperature (25 °C and 35 °C) levels, whereas the experiment with Delhi soil had no temperature and moisture factors; they were incubated at 25 °C and field capacity only. Air-dried soil weighing 150 g was mixed with 100 mg of NCPCs and placed in a beaker. The beakers were kept in a glass jar along with 0.1N NaOH solution to act as an alkali trap for evolved CO_2_. The excess NaOH was back titrated with HCl for estimation of C mineralization periodically at 7, 14, 21, 28, and 56th day of incubation [49]. The released CO_2_–C was quantified using the following equation:

1 mL of 1N NaOH = 6 mg of CO_2_–C

As we did in the release study, a set of blank soil samples without NCPC molecules was maintained to eliminate CO_2_ evolved only from soil samples.

### 2.7. Statistical Analysis

One-way analysis of variance (ANOVA) was performed on the collected data in accordance with a completely randomized design in order to examine variations in the treatment means, as expounded by Gomez and Gomez [50]. All the data analyses were performed using RStudio software (RStudio, PBC, Version 2024.04.2-764, Boston, MA, USA).

## 3. Results

### 3.1. FTIR Spectroscopy

**Figure 2:**
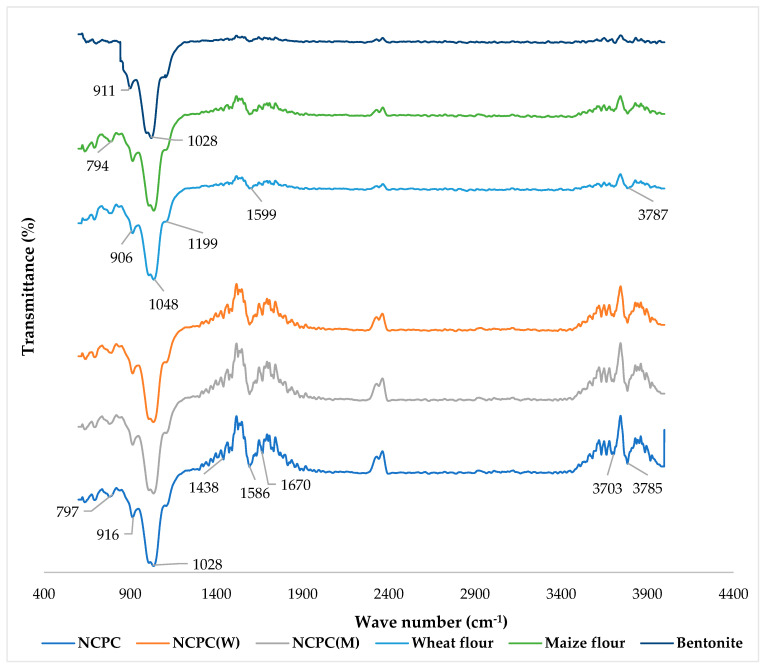
The FTIR spectra of different nanoclay polymer composites and raw materials.

The characteristic peaks of bentonite at 1028 cm^−1^ represent the Si–O stretching, and the peaks at 911 cm^−1^ are representative of the –OH group (Figure 2). The raw wheat and maize flour displayed the band at 3787 cm^−1^, which is attributed to O–H stretching in hydroxyl functional groups and the band at 1048 cm^−1^ is attributed to C–O stretching vibrations. The skeletal C=C stretching vibrations in the aromatic ring bands are responsible for the bands present in the 1640–1430 cm^−1^ range. The IR spectra of NCPCs show the presence of polyacrylic acid as indicated by the absorption bands at 1670 cm^−1^ and 1586 cm^−1^ due to –COOH stretching and asymmetric –COO– stretching, respectively. The bands near 1440 cm^−1^ (the skeletal C=C stretching vibrations in the aromatic rings) indicate the presence of wheat/maize starch in composite, while the peaks at 1028 cm^−1^ (of Si–O stretching) show the presence of bentonite. Thus, the resulting product was a composite based on poly acrylic acid + acrylamide incorporating bentonite and starch.

### 3.2. XRD Analysis

**Figure 3:**
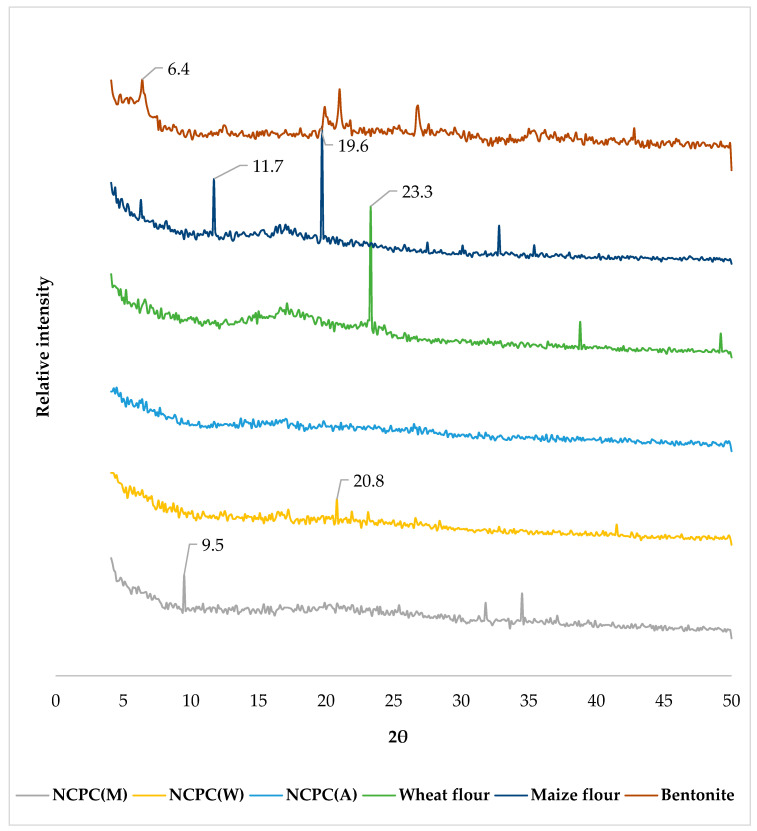
X-ray diffraction pattern of different nanoclay polymer composites and raw materials.

The mineralogy of bentonite and polymerization of NCPCs were confirmed using the XRD technique. The XRD patterns of the bentonite confirmed the presence of montmorillonite as a major phase as the peak at 2θ=6.4 (d spacing = 13.8 Ǻ) corresponds to smectite (2:1) minerals (Figure 3). The XRD pattern of NCPCs did not show the characteristic basal peak of bentonite particles after their incorporation into the polymer matrix. The crystalline nature of wheat and maize flour can be seen as diffraction peaks observed at 23.3 and 19.6 degrees 2θ, respectively, but in XRD patterns of NCPC(W) and NCPC(M) products, the characteristic peak of wheat and maize flour was very weak which shows the participation of wheat and maize starch in graft polymerization reaction with acrylic acid and acrylamide.

### 3.3. Water Absorption and Retention Capacity of Polymers

**Figure 4:**
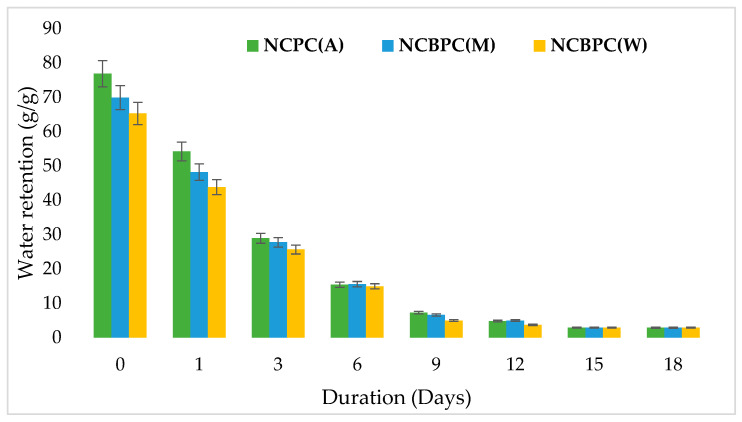
Water retention capacity of (NCPC(A), NCPC(M) and NCPC(W) over the incubation time. Vertical bars stand for the standard error (SE±).

It was observed that all the synthesized polymers reached their maximum water absorption capacities after 20 to 24 h. The maximum water absorbency was observed in NCPC(A) (76.86 g/g), followed by NCPC(M) (69.9 g/g) and NCPC(W) (65.27 g/g) (Table 1). The water retention capacity of the NCPCs over the duration is presented in Figure 4. The NCPCs gradually released the absorbed moisture, and after 15 to 18 days of incubation, polymers reached a moisture level that was in equilibrium with atmospheric moisture contents, and no further reduction in the moisture content was recorded. On the 18th day of incubation, the moisture content of the NCPC(A), NCPC(M), and NCPC(W) was 2.84 g/g, 2.84 g/g, and 2.85 g/g, respectively, which does not differ significantly.

### 3.4. Nitrogen Release in Soil

**Figure 5:**
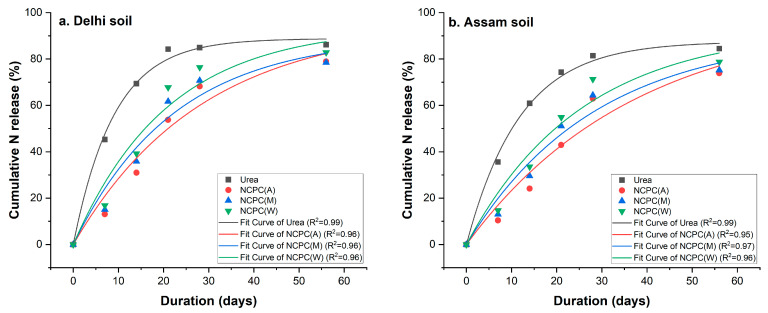
Effect of different N sources on cumulative inorganic N release in Delhi (**a**) and Assam (**b**) soils. The N release behaviour was predicted using the first-order kinetic equation, which is the best-fitted kinetic equation for the experimental data.

All three NCPCs showed significantly slower N release compared to urea and significantly affected the ammoniacal and nitrate N release in soil. In Delhi, soils (pH = 7.9), statistically significant (*p* = 0.05) higher values of NH_4_^+^–N contents were observed in NCPC(A) (38.92%), NCPC(M) (39.11%) and NCPC(W) (38.80%) as compared to urea (21.11%) at 56th day of incubation (Table 3). Significantly (*p* = 0.05) less NO_3_ content was observed in all treatments (NCPC(A):13.11%, NCPC(W) 39.33% and NCPC(M):44.0%) as compared to urea (65.05%) at the end of the incubation period. On the 56th day of incubation, the total mineral N release was in the following order: urea (86.16%) > NCPC(W) (82.80%) > NCPC(A) (78.93%) > NCPC(M) (78.44%) (Figure 5). Under Assam soils, the same pattern of NH_4_^+^-N release was observed, but the amount of released NH_4_^+^–N was less than in Delhi soil throughout the incubation period (Table 4). In Assam soils, the total mineral N release was in the following order: urea (35.62–84.47%) > NCPC(W) (14.66–78.76%) > NCPC(M) (13.08–75.24%) > NCPC(A) (10.40–73.92%) from 7th day to 56th day of incubation (Figure 5). Overall, among the NCPCs, the slowest rate of N release was recorded in NCPC(A), followed by NCBPC(M) and NCBPC(W) up to 56th days of incubation. Initially, the major portion of the released mineral N was contributed by NH_4_^+^–N, which started reducing after 28 days of incubation. Whereas the NO_3_–N, as well as total mineral N, keep increasing in the medium throughout the incubation period.

The release kinetics of urea and NCPC fertilizers were studied with zero-order, first-order, Higuchi, and Korsmeyer−Peppas models using linear curve fitting. The slope and intercept values derived from the linear equation were used to calculate the parameters given in Table 5. For all products, the results reveal that the linearized versions of the first-order, Higuchi, and Korsmeyer-Peppas models fit the data well, with R^2^ > 0.80. According to the R^2^ values obtained, the first-order kinetic model, which demonstrates the nutrient release depending on the concentration, gave the highest R^2^ for all the N fertilizers studied under both soils. In Delhi soils, the R^2^ values range between 0.96 and 0.99 across the fertilizer products for the first-order kinetic model (Figure 5). The first-order rate constant (*k*_1_) for urea, NCPC(A), NCPC(M), and NCPC(W) were 0.11, 0.04, 0.04, and 0.05, respectively, showing the slower N release rate in NCPCs as compared to urea. The maximum potential mineralizable N (*C*_0_) under different fertilizers was in the following order: NCPC(A) (95.6%) > NCPC(W) (93.9%) > NCPC(M) (89.8%), and urea (88.8%). In Assam soils, the R^2^ values range between 0.95 and 0.99 across the fertilizer products. The trend for k_1_ and *C*_0_ values of different fertilizers in Assam soils remains similar to Delhi soils (Table 5).

#### 3.4.1. Effect of Soil Type on N Release

**Figure 6:**
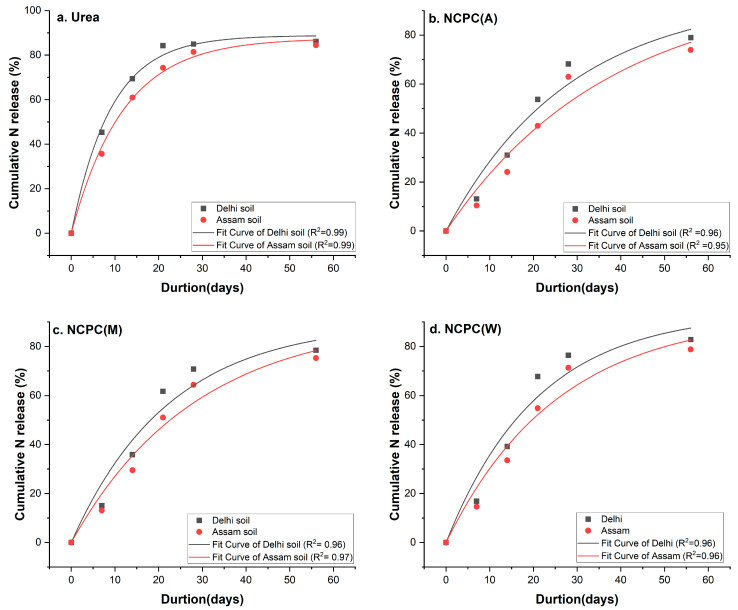
Effect of soil types on cumulative inorganic N release from urea (**a**), NCPC(A) (**b**), NCPC(M) (**c**) and NCPC(W) (**d**). The N release has been predicted using the first-order kinetic equation.

The effect of soil properties on N release kinetics was studied by incubating the fertilizer products with Delhi and Assam soils at FC moisture content and 25 °C temperature. The typical release rate of N from urea and NCPCs loaded with urea differed among the examined soils. The N release rates are slower in the Assam soils, which can be observed from the N release pattern of urea, NCPC(A), NCPC(W) and NCPC(M) in Figure 6. At the end of the incubation experiment, 6.77%, 4.23%, 5.12% and 2% higher cumulative N release was recorded from NCPC(A), NCPC(M), NCPC(W) and urea, respectively, in Delhi soils as compared to Assam soils. On close observation of Figure 6, one can notice that the difference in N release rate between the soils is more clearly visible at medium stages (14 to 28 days) as compared to the end of the incubation period. The maximum mineralizable N (*C*_0_) in Delhi soil ranges between 88.8 and 95.6 across the fertilizer products for the first-order kinetic model, whereas in Assam soils, these values vary from 87.6 to 99.2.

#### 3.4.2. Effect of Temperature on N Release

**Figure 7:**
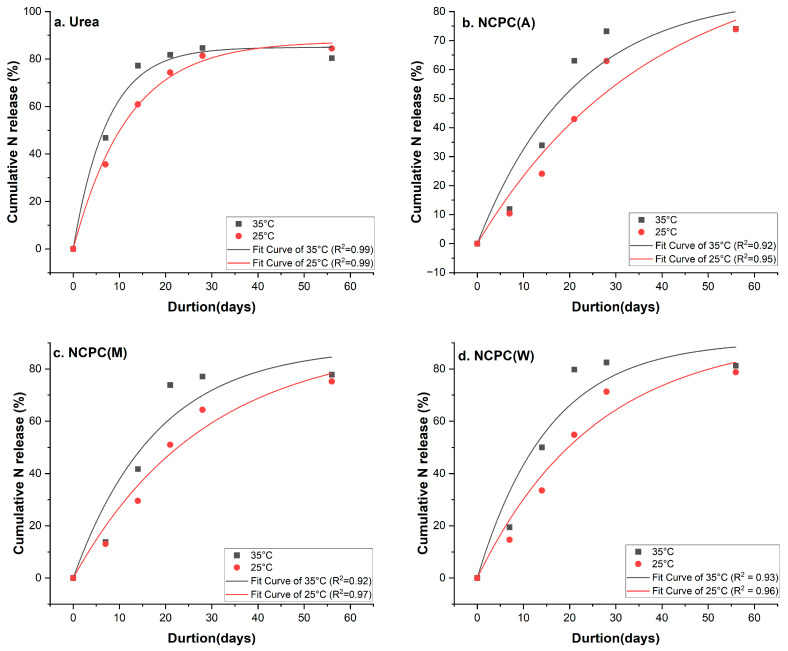
Effect of soil temperature on cumulative inorganic N release from urea (**a**), NCPC(A) (**b**), NCPC(M) (**c**) and NCPC(W) (**d**). The N release has been predicted using the first-order kinetic equation.

To investigate the effect of temperature on N release in soil, the fertilizer products with Assam soil were incubated at 25 °C and 35 °C temperature (Figure 7). At high temperature (35 °C), higher cumulative N release was recorded up to 28 days of incubation as compared to low temperature (25 °C). On the 28th day of incubation, the cumulative N release from NCPC(A), NCPC(M), NCPC(W) and urea was recorded at 62.92%, 64.39%, 71.28%, and 81.41%, respectively, at 25 °C, and 73.21%, 77.10%, 82.53%, and 84.7% of applied N, respectively at 35 °C. However, on the 56th day of incubation, the total mineral N content in the soil decreased slightly at 35 °C, whereas the N content at 25 °C kept increasing. Overall, increasing the temperature increased the N release from polymer-based fertilizers and conventional urea, which is also indicated by higher K_1_ values for N release at 35 °C than 25 °C. However, high temperature continuously for a longer duration may lead to losses of released mineral N from the medium, which is indicated by lower potential mineralizable N at higher temperatures (Table 5).

#### 3.4.3. Effect of Soil Moisture on N Release

**Figure 8:**
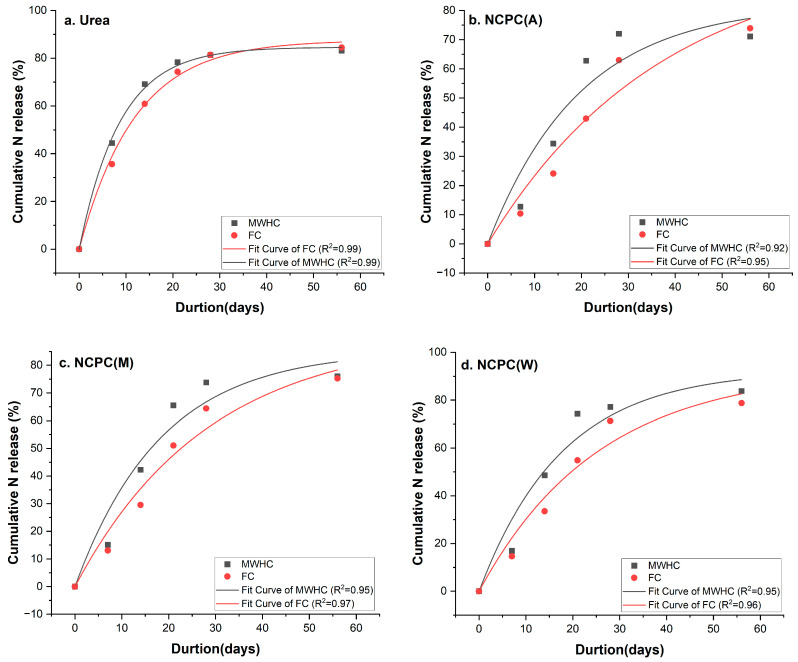
Effect of soil moisture on cumulative inorganic N release from urea (**a**), NCPC(A) (**b**), NCPC(M) (**c**) and NCPC(W) (**d**). The N release has been predicted using the first-order kinetic equation.

The effect of soil moisture contents (FC and MWHC) on N release rates of NCPCs and urea fertilizer is presented in Figure 8. The N release rates of NCPCs were significantly affected by soil moisture contents, especially from the 14th to 28th days of incubation, with a higher cumulative N release rate recorded at MWHC than FC moisture content. However, on the 56th day of incubation, the difference was not significant except in NCPC(W). At the end of the incubation, cumulative mineral release from urea, NCPC, NCPC(M), and NCPC(W) was 83.18%, 71.10%, 76.01%, and 83.79%, respectively, at MWHC moisture, whereas at FC moisture content it was 84.47%, 73.92%, 76.01% and 78.76%, respectively. Overall, the data suggest that soil moisture was one of the factors that explain differences in N release rates from different fertilizer products, but the differences were narrowed down at the later stages of the incubation.

### 3.5. Degradation of NCPCs in Soil

**Figure 9:**
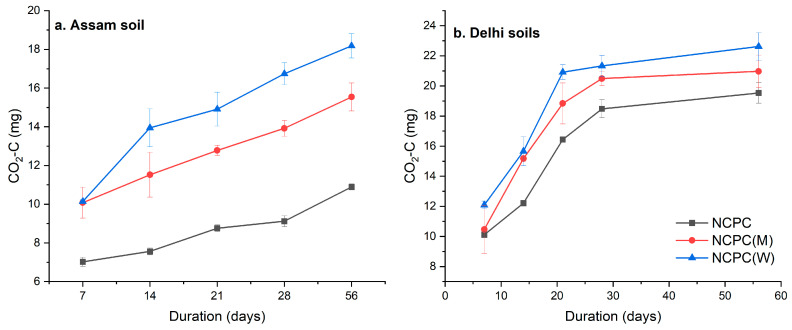
Cumulative CO_2_–C (mg/g) evolution from NCPC(A), NCPC(W) and NCPC(M) in Assam (**a**) and Delhi (**b**) soils.

Partial replacement of acrylic acid with the sources of starch (wheat/maize flour) for the synthesis of biodegradable NCPCs significantly improved the degradability of the fertilizer products in soil. Across the incubation time interval, the cumulative CO_2_–C evolution followed the trend as NCPC(W) > NCPC(M) > NCPC(A) under both Delhi and Assam soil (Figure 9). As the incubation time progressed from 7 to 56 days, the cumulative CO_2_–C evolution from the polymer composites increased. At the end of incubation duration, cumulative CO_2_–C evolution from NCPC(A), NCPC(M), and NCPC(W) were 10.89, 15.54 and 18.18 mg g^−1^, respectively, in Assam soil, whereas 19.53, 20.97, and 22.62 mg g^−1^, respectively, in Delhi soil. Overall, the order of cumulative CO_2_–C evolution, i.e., rates of degradation of NCPCs, was NCPC(W) > NCPC(M) > NCPC.

#### 3.5.1. Effect of Soil

**Figure 10:**
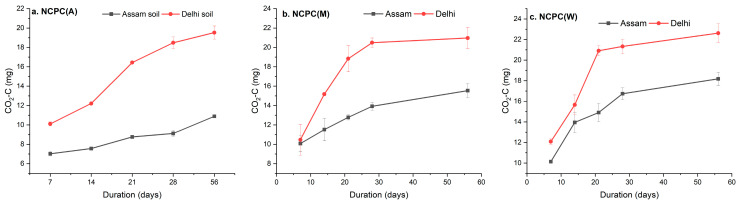
Effect of soil types on cumulative CO_2_–C (mg/g) evolution from NCPC(A) (**a**), NCPC(M) (**b**) and NCPC(W) (**c**). Vertical bars stand for the standard error (SE±).

Soil properties significantly affected the degradation rate of the polymer composites in soil. In Delhi soils (pH = 7.9), the evolution of CO_2_–C was significantly higher as compared to those obtained in Assam soil (pH = 4.2), regardless of the incubation time interval and fertilizer products. At 56 days after incubation, the cumulative CO_2_–C evolution was 19.53 mg to 22.62 mg in Delhi soil and 10.54 mg to 18.18 mg in Assam soil under different treatments (Figure 10).

#### 3.5.2. Effect of Temperature

**Figure 11:**
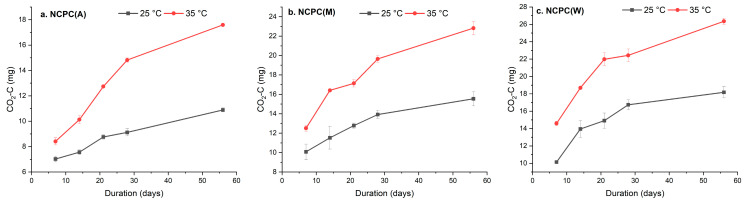
Effect of temperature on cumulative CO_2_–C (mg/g) evolution from NCPC(A) (**a**), NCPC(M) (**b**) and NCPC(W) (**c**) in Assam soil. Vertical bars stand for the standard error (SE±).

The increasing temperature significantly increased the degradation rate of polymer-based fertilizers, which can be explained by increased CO_2_–C evolution from polymers with increased temperature. At 35 °C temperature, the cumulative CO_2_–C evolution was enhanced by 30.11 to 66.10% as compared to 25 °C, irrespective of treatments at the end of the experiment (Figure 11). At 56th day of incubation the cumulative CO_2_–C evolution from NCPC(A), NCPC(M), and NCPC(W) were 10.89, 15.54, and 18.18 mg g^−1^, respectively, at 25 °C, whereas 17.59, 22.82, and 26.36 mg g^−1^, respectively, at 35 °C temperature.

#### 3.5.3. Effect of Soil Moisture

**Figure 12:**
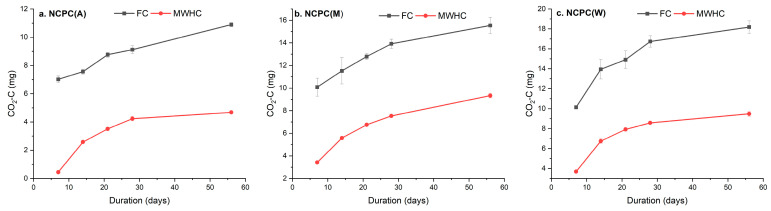
Effect of soil moisture on cumulative CO_2_-C (mg/g) evolution from NCPC(A) (**a**), NCPC(M) (**b**) and NCPC(W) (**c**) in Assam soil. Vertical bars stand for the standard error (SE±).

Soil moisture contents significantly affected the evolution of CO_2_–C from NCPC, NCPC(M) and NCPC(W) throughout the incubation period (Figure 12). The cumulative evolution of CO_2_–C was higher at FC moisture content than MWHC. On the 56th day of incubation, the cumulative evolution of CO_2_–C from NCPC(A), NCPC(M) and NCPC(W) was 4.68 mg. 9.33 mg and 9.48 mg g^−1^, respectively, at MWHC, whereas 10.89, 15.54, and 18.18 mg g^−1^ at FC soil moisture content. The data suggest that soil moisture is also one of the key factors that explain differences in the degradation rates of different fertilizer products.

## 4. Discussion

### 4.1. FTIR Spectroscopy

After the formation of NCPCs, the graft polymerization process between clay and monomers, as well as the dispersion of clay into the polymer matrix, caused the absorption peaks at 911 cm^−1^ of bentonite (caused by the –OH group) to disappear. However, the absorption peaks of Si–O were weakened but still present in NCPCs, showing the presence of bentonite [27,51]. Liang and Liu [32] also reported the graft copolymerization between the –OH groups on kaolin and the monomers during the polymerization reaction. In NCPC(M) and NCPC(W), the bands in the region 1640–1430 cm^−1^ are ascribed to the skeletal C=C stretching vibrations in the aromatic ring bands, indicating the existence of wheat/maize starch in the composite. The characteristic peaks of starch in starch/poly (acrylic acid-*co*-acrylamide) superabsorbent near 1650 of symmetric and 1159.07–992.53 cm^−1^ for asymmetric stretching vibrations of the C–O–C bridge. The symmetric stretching vibration of the C–O–C bridge caused a stronger and larger peak to develop at around 1670 cm^−1^ [52].

### 4.2. XRD Analysis

The characteristic peak of bentonite observed at 6.4 corresponds to the periodicity of the montmorillonite (MMT) in the direction of (001) [53]. This peak disappeared after polymerization, indicating an exfoliated structure. An exfoliated or delaminated structure is obtained when the silicate layers are completely and uniformly dispersed in a continuous polymer matrix. Due to either an excessively large spacing between the layers or the absence of ordering in the nanocomposite, no more diffraction peaks can be seen in the XRD diffractograms in exfoliated structures [54,55]. As the clay was added during the in-situ polymerization, polymer chains diffuse into the interlayer spaces of the silicate clays, which separates the clay layers and leads to partial or complete exfoliation, where individual silicate layers are dispersed within the polymer matrix [56]. Therefore, the XRD pattern of NCPCs did not show the characteristic peak of bentonite. Leitão et al. [57] reported that clay particles dispersed in the nanocomposite matrix due to the H-bond formation between the amide and acid groups present on the polymer matrix and the surrounding water molecules of the exchangeable cations on clay particles. In wheat and maize flour grafted NCPCs, the peaks disappeared or weakened. The crystalline portion of the starch may have also taken part in the graft polymerization event, as evidenced by the weakening and disappearance of the starch peaks [58,59].

### 4.3. Water Absorption and Retention Capacity of NCPCs

The NCPC(A) prepared without starch resulted in more water absorption and retention as compared to starch-grafted NCPCs, i.e., NCPC(M) and NCPC(W) that were prepared with maize and wheat starch, respectively. The dense polymeric network and a greater number of phenolic groups present in poly[(acrylic acid)-*co*-acrylamide] based NCPC (i.e., without starch) and compact structure and smaller three-dimensional net hole in starch-grafted-poly[(acrylic acid)-*co*-acrylamide] based NCPCs may be the reason for this phenomenon [52]. Similar findings were also reported by Saurabh [60], where AA+Am-based NCPC and sodium alginate grafted NCPC absorbed more water as compared to wheat starch-based NCPC. Liang et al. [61] have reported that water absorbency of the AA+Am NCPC decreases as the amount of starch in the polymer increases beyond 20%. The water absorbency decreased may be due to the fact that wheat starch powder was partly physically filled in the polymer network because the content of hydrophilic groups was lower, and the water absorbency thus gradually decreased.

### 4.4. N Release and Degradation of NCPCs

In our study, we obtained faster mineral N release from conventional urea-incubated soil samples over NCPC-treated soil. The primary reason behind this N availability is the higher solubility of urea in soil solution. The addition of urea to soil resulted in its rapid dissolution in soil solution followed by hydrolysis and subsequent conversion to ammoniacal form. This quicker hydrolysis and dissolution are, therefore, the cause of the increased mineral N concentration seen in urea during the early phases of the incubation (Table 3 and Table 4; Figure 5). However, in the case of NCPCs, urea was gradually released into the soil in exchange for free water, which impedes the hydrolysis of urea in an indirect manner. The slow N-release properties of superabsorbent polymer composites were also observed by Zhang et al. [62] and Sarkar et al. [5]. The cumulative NH_4_^+^–N release decreased over time however, the NO_3_^−^–N release increased since NO_3_^−^ is a transformation product of NH_4_^+^ via nitrification [63]. In the present study, N release was at a faster rate in NCPC(W) followed by NCPC(M) and lowest in NCPC(A). It might be due to less swelling of NCPC(W) and NCPC(M) (Table 3 and Table 4). Reduced swelling of polymers results in less absorption of urea inside the polymer and more urea adsorbed on the polymer’s surface, which releases in soil rapidly. Rapid degradation of starch-grafted NCPCs in soil may also be the cause of the quick release of N from NCPC(W) and NCPC(M) to soil [60,64].

Based on the results obtained from the fitting of different kinetic models, it is evident that three models, *viz*. first-order, Higuchi, and Korsmeyer−Peppas models, provided a good fit to the data for all the fertilizer products showing R*^2^* > 0.80. However, the first-order kinetic model most accurately describes the N release from both urea and NCPC-based N fertilizers, which is indicated by the consistently highest R² values in all the N fertilizer products irrespective of soil types. The first-order kinetic model indicated that the N release from the fertilizer products is concentration-dependent [45]. The hydrogel nanocomposite absorbs water slowly through diffusion and dissolves the fertilizer present in the matrix. The dissolved fertilizer diffuses via the polymer composite’s enlarged matrix and water-filled pores, per the Fickian diffusion principle [65]. which follows the first-order kinetics of nutrient release. Comparable results were reported by Wei et al. [66], where they synthesized slow-release N fertilizer using potato starch, acrylic acid/acrylamide, and maleic anhydride-modified cyclodextrin and observed that the first-order model, which demonstrates the urea release depending on the concentration, is the best appropriate model (R^2^ > 0.96) for the release of N.

Degradation of added polymer composites occurs in soil when microbes break down the polymer chains into CO_2_, water, and biomass following the initial chain scission cycle [67,68]. Hence, one can determine the extent of aerobic biodegradation of polymers by tracking the evolution of CO_2_ over time when the polymers were exposed to soil [69,70]. In this study, starch addition improved the biodegradability of the NCPCs, with NCPC(W) showing the highest cumulative CO_2_–C evolution (18.18–22.62 mg/g), followed by NCPC(M) (15.54–20.97 mg/g) and NCPC(A) (10.89–19.53 mg/g), this might be due to presence of more labile C in wheat and maize starch. Baldrian et al. [71] reported that biopolymers are an excellent source of C for soil microorganisms and are highly biodegradable. Therefore, applying a biopolymer may lead to increased activity of soil microorganisms and enzymes, followed by an increased C mineralization, which results in higher CO_2_ efflux [64].

#### 4.4.1. Effect of Soil

Soil type significantly influenced the N release from the NCPC fertilizer products. The two soils in this experiment had varying pH levels (Assam soil, pH = 4.2 and Delhi soil, pH = 7.9), which most likely had an impact on the microbial activity of the soil. The prevailing consensus is that soils with greater pH and organic C contents have higher rates of N mineralization from fertilizers [72,73]. It has been reported that the low pH of the soil negatively affects urea hydrolysis in soil [72,74]. From the research work conducted in earlier decades, it was reported that soil with a pH of 6.7 has a rate of N release approximately one-third higher than soil with a pH of 5.5 [74]. In our experiment also, a slow rate of N release as well as CO_2_ evolution was recorded under low pH soils, i.e., Assam soil (pH = 4.2) as compared to Delhi soil (pH = 7.9). The rate of N release in Assam soils was slow, but the higher overall recovery of N was observed as indicated by higher *C*_0_ values in Assam soil as compared to Delhi soils, irrespective of fertilizers. Fan and Li [75] also reported that for successful implementation of N fertilizers in field crops, soil pH variations must be taken into account when modifying the fertilizer composition or application rates.

#### 4.4.2. Effect of Temperature

The N release rates for urea, NCPC, NCPC(M) and NCPC(W) increased with increasing temperature. Since urease activity rises with temperature between 10 °C and 40 °C, we relate the temperature effect on urea to slower hydrolysis of urea at low temperatures [63,76]. In our investigation, the N release from the urea-loaded NCPCs proceeded rapidly with increasing incubation temperature, which might be due to rapid diffusion from the polymer fertilizers [77,78]. Temperature has a significant influence on the diffusion of dissolved urea from the polymer into soil solution because, in general, the diffusion rate increases (and lowers) with temperature [76,79]. Moreover, rapid hydrolysis of urea released from NCPCs at higher temperatures establishes a steeper concentration gradient, increasing diffusion out of the polymers [80]. Lower temperatures may, therefore, slow down the release of N, as the released N may accumulate around the polymers [81]. We, therefore, infer that low temperature reduced both the diffusion rate and the concentration gradient because of hydrolysis, causing a decrease in release rates. Another possible reason for the increased release rate of N may be the rapid degradation of polymers with increasing temperature. C mineralization increases significantly with the temperature and releases CO_2_–C when the polymer chains are metabolized by microorganisms [68].

#### 4.4.3. Effect of Moisture

The N release rate of all the synthesized NCPCs is strongly influenced by the moisture content of the soil at the initial days of incubation, but at the later stages of incubation, the effect is not clear (Figure 8). Soil water is not a limiting factor for plant nutrition at or above the FC moisture content [82]. But for urea to dissolve, diffuse, and undergo hydrolysis, soil water/moisture is necessary [79]. Reduced soil moisture could increase the lag time for N release from NCPC molecules. Golden et al. [76] also reported a lower initial N release at lower moisture levels when polymer-coated urea fertilizers were incubated in the soil at different moisture levels under controlled conditions. Several researchers mentioned the existence of a positive correlation between the nutrient release rate and degradation of polymers [27,64], but the condition might alter if moisture content varied. In the present investigation, the lower CO_2_ evolution, i.e., slow degradation but higher N release, was observed at higher soil moisture content. The lower CO_2_ evolution might be due to the reduced condition created at MWHC moisture content, which reduces the oxidation rate of C [83].

## 5. Conclusions

The findings of the present investigation offer valuable insights into the development and evaluation of starch-grafted-poly[(acrylic acid)-*co*-acrylamide] nanoclay polymer composites (NCPCs) as slow-release nitrogen (N) fertilizers. The results demonstrate that NCPC-based fertilizers, NCPC(A), NCPC(M), and NCPC(W), significantly slow down the N release compared to conventional urea. The slower release of N, which was well-described by the first-order kinetic model, highlights the ability of these composites to provide a sustained N delivery for an extended period, making them promising alternatives to conventional N fertilizers. Incorporating wheat and maize starch into the poly[(acrylic acid)-*co*-acrylamide] matrix improved the biodegradability of the NCPCs, which is reflected by the higher cumulative CO_2_–C evolution in starch-grafted NCPCs, i.e., NCPC(W) and NCPC(M) compared to NCPC(A). This improvement in biodegradability is crucial for addressing environmental concerns associated with synthetic polymers in fertilizers. Soil properties, including pH and moisture content and environmental factors such as temperature, significantly influenced the N release and degradation rates of NCPCs. The release of N was slower in Assam soils (acidic) compared to Delhi soils (alkaline), while higher temperatures accelerated both N release and microbial degradation. These findings underscore the importance of tailoring NCPC formulations to specific soil and climatic conditions to optimize their performance. Overall, the study demonstrates that starch-grafted NCPC-based fertilizers offer sustained N release, improved degradability, and reduced environmental impacts that would be useful for agricultural applications. Future research should focus on field trials to further explore their performance under diverse agricultural systems.

## Figures and Tables

**Table 1:** Characteristics of synthesized nanoclay polymer composite fertilizers.

Fertilizer Product	Polymer Composition	% N Content	% C Content	Equilibrium Water Absorbency (g/g)
NCPC(A)	Poly[(acrylic acid)-*co*-acrylamide]	32.28	26.38	76.86
NCPC(M)	Maize starch-grafted-poly[(acrylic acid)-*co*-acrylamide]	31.06	27.09	69.90
NCPC(W)	Wheat starch-grafted-poly[(acrylic acid)-*co*-acrylamide]	32.32	25.35	65.27

**Table 2:** Important physico-chemical properties of the experimental soils.

Parameter	Reference	Typic Haplustepts (Delhi Soil)	Typic Hapludults (Assam Soil)
pH (1:2.5)	Jackson [36]	7.9	4.2
EC (1:2.5) (dS m^−1^)	Jackson [36]	0.32	0.09
Organic carbon (%)	Walkley and Black [37]	0.41	0.67
CEC [cmol(p+) Kg^−1^]	Bouyoucos [38]	12.2	7.6
Mechanical composition			
Sand (%)		65.1	45.5
Silt (%)		19.6	9.8
Clay (%)		15.3	44.7
Texture		Sandy loam	Sandy clay
Available N (kg ha^−1^)	Subbiah and Asija [39]	189	339
Available P (kg ha^−1^)	Olsen et al. [40]; Bray and Kutz [41]	20.3	21.6
Available K (kg ha^−1^)	Hanway and Heidel [42]	292	186

**Table 3:** Effect of Different N Sources on NH_4_^+^− N and NO_3_^−^− N Release in Delhi Soils.

Treatment	NH_4_^+^− N (%)					NO_3_^−^− N (%)				
	7 Days	14 Days	21 Days	28 Days	56 Days	7 Days	14 Days	21 Days	28 Days	56 Days
**Urea**	31.66 ^a^	39.58 ^a^	42.51 ^a^	38.56 ^b^	21.11 ^b^	13.66 ^a^	29.81 ^a^	41.76 ^a^	46.37 ^a^	65.05 ^a^
**NCPC(A)**	8.58 ^c^	18.43 ^c^	34.41 ^d^	39.80 ^b^	38.92 ^a^	4.53 ^c^	12.54 ^d^	19.35 ^d^	28.40 ^d^	40.01 ^c^
**NCPC(W)**	10.87 ^b^	21.95 ^b^	41.07 ^b^	42.73 ^a^	38.80 ^a^	5.98 ^b^	17.27 ^b^	26.69 ^b^	33.68 ^b^	44.00 ^b^
**NCPC(M)**	9.21 ^c^	19.96 ^c^	38.50 ^c^	40.00 ^b^	39.11 ^a^	5.84 ^b^	15.91 ^c^	23.20 ^c^	30.71 ^c^	39.33 ^c^
**LSD (*p* = 0.05)**	2.08	2.90	2.71	1.74	2.28	0.99	1.78	2.30	1.97	1.71
**SEm±**	0.64	0.89	0.83	0.53	0.70	0.30	0.55	0.68	0.60	0.52

Values in a column that are followed by the same letter do not differ substantially but the values followed by different letters differ significantly at *p* < 0.05

**Table 4:** Effect of different N Sources on NH_4_^+^− N and NO_3_^−^− N Release in Assam Soils.

Treatment	NH_4_^+^− N (%)					NO_3_^−^− N (%)				
	7 Days	14 Days	21 Days	28 Days	56 Days	7 Days	14 Days	21 Days	28 Days	56 Days
**Urea**	24.65 ^a^	33.98 ^a^	37.57 ^a^	38.51 ^ab^	29.11 ^b^	10.97 ^a^	26.91 ^a^	36.76 ^a^	42.90 ^a^	55.37 ^a^
**NCPC(A)**	6.76 ^c^	14.43 ^d^	25.61 ^c^	35.98 ^c^	35.92 ^a^	3.64 ^c^	9.68 ^c^	17.33 ^c^	26.94 ^c^	38.00 ^c^
**NCPC(W)**	8.87 ^b^	20.96 ^b^	32.89 ^b^	39.60 ^a^	37.76 ^a^	5.78 ^b^	12.59 ^b^	21.97 ^b^	31.67 ^b^	41.00 ^b^
**NCPC(M)**	7.92 ^bc^	17.60 ^c^	31.05 ^b^	36.68 ^bc^	37.11 ^a^	5.15 ^b^	11.96 ^b^	20.00 ^b^	27.71 ^c^	38.13 ^bc^
**LSD (*p* = 0.05)**	1.97	2.17	2.01	2.52	2.15	0.82	1.68	2.40	2.58	2.88
**SEm±**	0.61	0.66	0.61	0.77	0.66	0.25	0.51	0.74	0.79	0.88

Values in a column that are followed by the same letter do not differ substantially but the values followed by different letters differ significantly at *p* < 0.05

**Table 5:** Parameter Estimates of the Kinetic Models for the Inorganic N Release From Urea, NCPC(A), NCPC(M) and NCPC(W) N Fertilizers.

Soil Type	Temp.	Moisture	Fertilizer	Kinetic Parameters										
				Zero Order			First Order			Korsmeyer-Peppas			Higuchi	
				*Q*_0_	*k*_0_	R^2^	*C*_0_	*k*_1_	R^2^	*k*	*n*	R^2^	*k_H_*	R^2^
Delhi soil	25 °C	FC	Urea	35	1.27	0.55	88.8	0.11	0.99	34.8	0.25	0.94	14.8	0.90
			NCPC(A)	10.4	1.45	0.84	95.6	0.04	0.96	7.16	0.62	0.92	10.7	0.92
			NCPC(M)	13.9	1.42	0.78	89.8	0.04	0.96	9.70	0.54	0.90	11.2	0.90
			NCPC(W)	15.9	1.49	0.76	93.9	0.05	0.96	11.2	0.52	0.89	12.1	0.89
Assam soil	25 °C	FC	Urea	28.2	1.33	0.64	87.6	0.10	0.99	24.7	0.33	0.94	13.7	0.90
			NCPC(A)	6.65	1.38	0.87	99.2	0.03	0.95	4.81	0.7	0.92	9.53	0.90
			NCPC(M)	10	1.37	0.84	87.7	0.03	0.97	6.86	0.61	0.93	10.2	0.92
			NCPC(W)	12	1.44	0.82	92.9	0.04	0.96	8.28	0.58	0.92	11	0.91
Assam soil	25 °C	MWHC	Urea	33.9	1.21	0.55	84.6	0.11	0.99	34.3	0.24	0.95	14.2	0.85
			NCPC(A)	14.6	1.31	0.70	82	0.05	0.92	4.81	0.7	0.75	10.8	0.84
			NCPC(M)	16.8	1.36	0.71	85.3	0.05	0.95	12.3	0.48	0.87	11.6	0.87
			NCPC(W)	19.1	1.48	0.71	92.5	0.06	0.95	14.0	0.47	0.88	12.7	0.87
Assam soil	35 °C	FC	Urea	38.1	1.13	0.45	85.0	0.13	0.99	41.6	0.19	0.92	14.6	0.77
			NCPC(A)	13.8	1.38	0.72	86.0	0.05	0.92	9.81	0.53	0.85	11.0	0.85
			NCPC(M)	17.6	1.42	0.67	88.5	0.06	0.92	12.9	0.48	0.83	12.1	0.83
			NCPC(W)	35.0	0.82	0.21	90.8	0.06	0.93	17.0	0.42	0.84	13.1	0.83

Assam soils with N sources were incubated at two soil moisture (field capacity (FC) and maximum water holding capacity (MHWC)) and temperature (25 and 35 °C) levels to study the effect of respective factors on N release. Delhi soils with N sources were incubated at 25 °C temperature and FC soil moisture only.

## Data Availability

Data are contained within the article.

## References

[1] Corradini E., De Moura M.R., Mattoso L.H.C. (2024). A preliminary study of the incorporation of NPK fertilizer into chitosan nanoparticles. Express Polym. Lett..

[2] Wu Z., Luo J., Han Y., Hua Y., Guan C., Zhang Z. (2019). Low nitrogen enhances nitrogen use efficiency by triggering NO_3_–uptake and its long-distance translocation. J. Agri. Food Chem..

[3] International Fertilizer Industry Association (IFA) (2021). Databases, Total N Consumptions. https://www.ifastat.org/databases/plant-nutrition.

[4] Fertilizer Association of India (FAI) (2024). Fertilizer Statistics. https://www.faidelhi.org/statistics/statistical-database.

[5] Sarkar A., Biswas D.R., Datta S.C., Dwivedi B.S., Bhattacharyya R., Kumar R., Patra A.K. (2021). Preparation of novel biodegradable starch/poly (vinyl alcohol)/bentonite grafted polymeric films for fertilizer encapsulation. Carbo. Polym..

[6] Linquist B.A., Adviento-Borbe M.A., Pittelkow C.M., van Kessel C., van Groenigen K.J. (2012). Fertilizer management practices and greenhouse gas emissions from rice systems: A quantitative review and analysis. Field Crops Res..

[7] Cookson W.R., Osman M., Marschner P., Abaye D.A., Clark I., Murphy D.V., Stockdale E.A., Watson C.A. (2007). Controls on soil nitrogen cycling and microbial community composition across land use and incubation temperature. Soil Biol. Biochem..

[8] Fan X., Li F., Liu F., Kumar D. (2004). Fertilization with a new type of coated urea: Evaluation for nitrogen efficiency and yield in winter wheat. J. Plant Nutr..

[9] Herrera J., Rubio G., Häner L., Delgado J., Lucho-Constantino C., Islas-Valdez S., Pellet D. (2016). Emerging and established technologies to increase nitrogen use efficiency of cereals. Agronomy.

[10] Omara P., Aula L., Oyebiyi F., Raun W.R. (2019). World cereal nitrogen use efficiency trends: Review and current knowledge. Agrosys. Geosci. Environ..

[11] Asghari H.R., Cavagnaro T.R. (2011). Arbuscular mycorrhizas enhance plant interception of leached nutrients. Funct. Plant Biol..

[12] Chatterjee D., Das S.R., Mohanty S., Muduli B.C., Bhatia A., Nayak B.K., Rees R.M., Drewer J., Nayak A.K., Adhya T.K. (2024). Reducing the environmental impact of rice production in subtropical India by minimising reactive nitrogen loss. J. Environ. Manag..

[13] Trenkel M.E. (2010). Slow- and Controlled-Release and Stabilized Fertilizers: An Option for Enhancing Nutrient Use Efficiency in Agriculture.

[14] Naz M.Y., Sulaiman S.A. (2016). Slow-release coating remedy for nitrogen loss from conventional urea: A review. J. Control Release.

[15] Azeem B., KuShaari K., Man Z.B., Basit A., Thanh T.H. (2014). Review on materials & methods to produce controlled release coated urea fertilizer. J. Control Release.

[16] McKenzie R.H., Bremer E., Middleton A.B., Pfiffner P.G., Dowbenko R.E. (2007). Controlled-release urea for winter wheat in southern Alberta. Can. J. Soil Sci..

[17] Galloway J.N., Cowling E.B. (2021). Reflections on 200 years of Nitrogen, 20 years later. Ambio.

[18] Menegat S., Ledo A., Tirado R. (2022). Greenhouse gas emissions from global production and use of nitrogen synthetic fertilizers in agriculture. Sci. Rep..

[19] Kumar A., Manjaiah K.M., Sharma V.K., Chobhe K.A., Suman A., Bhatia A., Saini R., Meena S., Kumar A.N., Roy D. (2024). Evaluation of urea loaded nanoclay biopolymer composites with Zn and P solubilizing microbes for nitrogen uptake and use efficiency in maize (Zea mays)-wheat (Triticum aestivum) cropping system. Indian J. Agric. Sci..

[20] Gil-Ortiz R., Naranjo M.Á., Ruiz-Navarro A., Atares S., García C., Zotarelli L., San Bautista A., Vicente O. (2020). Enhanced agronomic efficiency using a new controlled-released, polymeric-coated nitrogen fertilizer in rice. Plants.

[21] Messiga A.J., Dyck K., Ronda K., van Baar K., Haak D., Yu S., Dorais M. (2020). Nutrients leaching in response to long-term fertigation and broadcast nitrogen in blueberry production. Plants.

[22] Hüttermann A., Orikiriza L.J.B., Agaba H. (2009). Application of superabsorbent polymers for improving the ecological chemistry of degraded or polluted lands. Clean.

[23] Seetapan N., Wongsawaeng J., Kiatkamjornwong S. (2011). Gel strength and swelling of acrylamide-protic acid superabsorbent copolymers. Polym. Adv. Technol..

[24] Chang L., Xu L., Liu Y., Qiu D. (2021). Superabsorbent polymers used for agricultural water retention. Polym. Test.

[25] Ali O., Hamid Z., Dariush S., Abdolreza M., Adel R.T. (2018). Slow-release NPK fertilizer encapsulated by carboxymethyl cellulose-based nanocomposite with the function of water retention in soil. Mater. Sci. Eng..

[26] Sarkar S., Datta S.C., Biswas D.R. (2015). Effect of fertilizer loaded nanoclay/superabsorbent polymer composites on nitrogen and phosphorus release in soil. Proc. Natl. Acad. Sci. India Sect. B Biol. Sci..

[27] Kirti S., Manjaiah K.M., Datta S.C., Biswas D.R., Arti B., Bandyopadhyay K.K., Ritu T. (2021). Mitigating nitrous oxide emission using nanoclay-polymer composites in rice-wheat cropping system. Arch. Agron. Soil Sci..

[28] Mohammadi N., Shariatmadari H., Khademi H., Bazarganipour M. (2020). Coating of sepiolite-chitosan nanocomposites onto urea increases nitrogen availability and its use efficiency in maize. Arch. Agron. Soil Sci..

[29] Wen Q., Chen Z., Zhao Y., Zhang H., Feng Y. (2010). Biodegradation of polyacrylamide by bacteria isolated from activated sludge and oil-contaminated soil. J. Hazard Mater..

[30] Kay-Shoemake J.L., Watwood M.E., Sojka R.E., Lentz R.D. (2000). Soil amidase activity in polyacrylamide-treated soils and potential activity toward common amide-containing pesticides. Biol. Fertil. Soils.

[31] Lakshani N., Wijerathne H.S., Sandaruwan C., Kottegoda N., Karunarathne V. (2023). Release Kinetic Models and Release Mechanisms of Controlled-Release and Slow-Release Fertilizers. ACS Agri. Sci. Technol..

[32] Liang R., Liu M. (2007). Preparation of poly (acrylic acid-*co*-acrylamide)/kaolin and release kinetics of urea from it. J. Appl. Poly. Sci..

[33] Bragg W.L. (1912). The diffraction of short electromagnetic waves by a crystal. Proc. Camb. Philos. Soc..

[34] Buresh R.J., Austin E.R., Craswell E.T. (1982). Analytical methods in 15N research. Fertil. Res..

[35] Singh A., Sarkar D.J., Singh A.K., Parsad R., Kumar A., Parmar B.S., Singh B.S. (2011). Studies on Novel Nanosuperabsorbent Composites: Swelling Behavior in Different Environments and Effect on Water Absorption and Retention Properties of Sandy Loam Soil and SoilLess Medium. J. Appl. Polym. Sci..

[36] Jackson M.L. (1973). Soil Chemical Analysis.

[37] Walkley A., Black I.A. (1934). An examination of the Degtjareff method for determining soil organic matter and a proposed modification of the chromic acid titration method. Soil Sci..

[38] Bouyoucos G.J. (1962). Hydrometer method improved for making particle size analysis of soils. Agron. J..

[39] Subbiah B.V., Asija G.L. (1956). Alkaline permanganate method. Curr. Sci..

[40] Olsen S.R. (1954). Estimation of Available Phosphorus in Soils by Extraction with Sodium Bicarbonate (No. 939).

[41] Bray R.H., Kurtz L.T. (1965). Determination of total, organic, and available forms of phosphorus in soils. Soil Sci..

[42] Hanway J.J., Heidel H. (1952). Soil analysis methods as used in Iowa State College Soil Testing Laboratory. Iowa Agricu..

[43] Keeney D.R., Nelson D.W., Page A.L., Miller R.H., Keeney D.R. (1982). Nitrogen Inorganic Forms. Methods of Soil Analysis. Agronomy Monograph 9 Part 2.

[44] Varelas C.G., Dixon D.G., Steiner C. (1995). Zero-order release from biphasic polymer hydrogels. J. Control Release.

[45] Agehara S., Warncke D.D. (2005). Warncke. Soil moisture and temperature effects on nitrogen release from organic nitrogen sources. Soil Sci. Soc. Am. J..

[46] Deans J., Molina J., Clapp C. (1986). Models for predicting potentially mineralizable nitrogen and decomposition rate constants. Soil Sci. Soc. Am. J..

[47] Higuchi T. (1963). Mechanism of sustained action medication: Theoretical analysis of rate of release of solid drugs dispersed in solid matrices. J. Pharm. Sci..

[48] Korsmeyer R.W., Gurny R., Doelker E., Buri P., Peppas N.A. (1983). Mechanisms of solute release from porous hydrophilic polymers. Int. J. Pharm..

[49] Zibilske L.M. (1994). Carbon mineralization. Methods of Soil Analysis: Part 2 Microbiological and Biochemical Properties.

[50] Gomez K.A., Gomez A.A. (1984). Statistical Procedures for Agricultural Research.

[51] Qin S., Wu Z., Rasool A., Li C. (2012). Synthesis and characterization of slow-release nitrogen fertilizer with water absorbency: Based on poly (acrylic acid-acrylic amide)/Na-bentonite. J. Appl. Polym. Sci..

[52] Jin S., Wang Y., He J., Yang Y., Yu X., Yue G. (2013). Preparation and properties of a degradable interpenetrating polymer networks based on starch with water retention, amelioration of soil, and slow release of nitrogen and phosphorus fertilizer. J. Appl. Polym. Sci..

[53] Wang S., Hu Y., Zhongkai Q., Wang Z., Chen Z., Fan W. (2003). Preparation and flammability properties of polyethylene/clay nanocomposites by melt intercalation method from Na^+^ montmorillonite. Mater. Lett..

[54] Pavlidou S., Papaspyrides C.D. (2008). A review on polymer-layered silicate nanocomposites. Prog. Polym. Sci..

[55] Alexandre M., Dubois P. (2000). Polymer-layered silicate nanocomposites: Preparation, properties and uses of a new class of materials. Mater. Sci Eng R Rep..

[56] Mittal V. (2009). Polymer layered silicate nanocomposites: A review. Materials.

[57] Leitão R.C., Moura C.P.D., da Silva L.R., Ricardo N.M., Feitosa J., Muniz E.C., Rodrigues F.H. (2015). Novel superabsorbent hydrogel composite based on poly (acrylamide-*co*-acrylate)/nontronite: Characterization and swelling performance. Química Nova.

[58] Gao J., Tian R., Yu J., Duan M. (1994). Graft copolymers of methyl methacrylate onto canna starch using manganic pyrophosphate as an initiator. Appl. Polym. Sci..

[59] Zou W., Liu X., Yu L., Qiao D., Chen L., Liu H. (2013). Synthesis and characterization of biodegradable starch-polyacrylamide graft copolymers using starches with different microstructures. J. Polym. Environ..

[60] Saurabh K. (2016). Nanoclay Polymer Composites (NCPCs) with Biodegradable Polymers for Controlled Release of Nitrogen in Rice and Wheat Crops. Ph.D. Thesis.

[61] Liang R., Yuan H., Xi G., Zhou Q. (2009). Synthesis of wheat straw-g-poly (acrylic acid) superabsorbent composites and release of urea from it. Carbo. Polym..

[62] Zhang B., Cui Y., Yin G., Li X., Liao L., Cai X. (2011). Synthesis and swelling properties of protein-poly (acrylic acid-*co*-acrylamide) superabsorbent composite. Polym. Comp..

[63] Sentek V., Velescu A., Wilcke W., Henke C., Peters N., Welp G., Amelung W. (2023). Nitrogen release from different polymer-coated urea fertilizers in soil is affected by soil properties. Soil Use Manag..

[64] Mandal N., Datta S.C., Manjaiah K.M., Dwivedi B.S., Kumar R., Aggarwal P. (2018). Zincated nanoclay polymer composites (ZNCPCs): Synthesis, characterization, biodegradation and controlled release behaviour in soil. Polym. Plast. Technol. Eng..

[65] Rashidzadeh A., Olad A., Reyhanitabar A. (2015). Hydrogel/ clinoptilolite nanocomposite-coated fertilizer: Swelling, water-retention and slow-release fertilizer properties. Polym. Bull..

[66] Wei H., Wang H., Chu H., Li J. (2019). Preparation and characterization of slow-release and water-retention fertilizer based on starch and halloysite. Int. J. Biol. Macromol..

[67] Moore G.F., Saunders S.M. (1998). Advances in Biodegradable Polymers.

[68] Premraj R., Doble M. (2005). Biodegradation of polymers. Indian J. Biotechnol..

[69] Modelli A., Calcagno B., Scandola M. (1999). Kinetics of aerobic polymer degradation in soil by means of the ASTM D 5988-96 standard method. J. Environ. Polym. Degrad..

[70] Calmon A., Dusserre-Bresson L., Bellon-Maurel V., Feuilloley P., Silvestre F. (2000). An automated test for measuring polymer biodegradation. Chemosphere.

[71] Baldrian P., Voříšková J., Dobiášová P., Merhautová V., Lisá L., Valášková V. (2011). Production of extracellular enzymes and degradation of biopolymers by saprotrophic microfungi from the upper layers of forest soil. Plant Soil.

[72] Tlustos P., Blackmer A.M. (1992). Release of nitrogen from ureaform fractions as infl uenced by soil pH. Soil Sci. Soc. Am. J..

[73] Gioacchini P., Ramieri N.A., Montecchio D., Marzadori C., Ciavatta C. (2006). Dynamics of mineral nitrogen in soils treated with slow-release fertilizers. Commun. Soil Sci. Plant Anal..

[74] Bredakis E.J., Steckel J.E. (1963). Steckel. Leachable nitrogen from soils incubated with turfgrass fertilizers. Agron. J..

[75] Fan X.H., Li Y.C. (2010). Nitrogen release from slow-release fertilizers as affected by soil type and temperature. Soil Sci. Soc. Am. J..

[76] Golden B., Slaton N., Norman R., Gbur E., Wilson C. (2011). Nitrogen release from environmentally smart nitrogen fertilizer as influenced by soil series, temperature, moisture, and incubation method. Commun. Soil Sci. Plant Anal..

[77] Medina L.C., Sartain J.B., Obreza T.A., Hall W.L., Thiex N.J. (2014). Evaluation of a soil incubation method to characterize nitrogen release patterns of slow- and controlled-release fertilizers. J. AOAC Int..

[78] Trolove S., Wijeyekoon S., Tan Y., Currie L.D., Christensen C.L. (2019). Effect of Temperature on the Rate of Nitrogen Release from a Controlled Release Fertilizer. Nutrient Loss Mitigations for Compliance in Agriculture. Occasional Report.

[79] Ransom C.J., Jolley V.D., Blair T.A., Sutton L.E., Hopkins B.G. (2020). Nitrogen release rates from slow- and controlled-release fertilizers influenced by placement and temperature. PLoS ONE.

[80] Carson L.C., Ozores-Hampton M. (2013). Factors affecting nutrient availability, placement, rate, and application timing of controlled-release fertilizers for Florida vegetable production using seepage irrigation. HortTechnology.

[81] Lunt O.R., Oertli J.J. (1962). Controlled release of fertilizer minerals by incapsulating membranes: II. Efficiency of recovery, influence of soil moisture, mode of application, and other considerations related to use. Soil Sci. Soc. Am. J..

[82] Cahill S., Osmond D., Israel D. (2010). Nitrogen release from coated urea fertilizers in different soils. Commun. Soil Sci. Plant Anal..

[83] Banerjee B., Aggarwal P.K., Pathak H., Singh A.K., Chaudhary A. (2006). Dynamics of organic carbon and microbial biomass in alluvial soil with tillage and amendments in rice-wheat systems. Environ. Moni. Assess..

